# Case report: A diabetic patient with cryptococcal meningoencephalitis complicated by post-infectious inflammatory response syndrome

**DOI:** 10.3389/fimmu.2024.1444486

**Published:** 2024-11-27

**Authors:** Qinghua Chen, Weitong Yu, Xuyi Wang, Chenxi Zhao, Pin Wang, Lin Sun, Linlin Xu, Yingying Xu

**Affiliations:** ^1^ The Second Hospital of Shandong University, Cheeloo College of Medicine of Shandong University, Shandong University, Jinan, China; ^2^ Department of Neurology Medicine, The Second Hospital of Shandong University, Cheeloo College of Medicine of Shandong University, Shandong University, Jinan, China

**Keywords:** cryptococcal meningoencephalitis (CM), diabetes mellitus (DM), ommaya capsule, post-infectious inflammatory response syndrome (PIIRS), human immunodeficiency

## Abstract

We report on a previously non-HIV-diagnosed, 47-year-old male diagnosed with diabetes mellitus (DM) and cryptococcal meningoencephalitis, who was referred to our institution for antifungal treatment. During the course of treatment, due to the development of refractory intracranial hypertension, Ommaya reservoirs were employed for cranial pressure reduction. The patient gradually recovered during subsequent antifungal therapy; however, symptoms worsened in the third month of treatment, leading to consideration of post-infectious inflammatory response syndrome (PIIRS) on examination. Once diagnosed, the symptoms improved significantly after approximately 130 days of treatment with additional corticosteroids.

## Introduction

In adults, fungal meningitis is predominantly characterized by cryptococcal meningitis across much of the world (1, 2), which is associated with high morbidity and mortality. On an international scale, it is generally categorized into HIV-diagnosed and non-HIV-diagnosed CM (cryptococcal meningoencephalitis) patients according to treatment regimen. In people living with HIV, when patients are put on antiretroviral therapy, the excessive immune response observed during treatment is termed immune reconstitution inflammatory syndrome (IRIS) (3); for non-HIV-diagnosed patients, a decline in neurological status following the commencement of antifungal therapy is attributed not to the therapy’s failure but to an enhanced immune response, a condition termed PIIRS (2, 4).

Type II diabetes is a metabolic disorder characterized by chronic hyperglycemia. China harbors the largest population of Type II diabetic patients globally, with approximately 11.6% diagnosed with diabetes and 50.1% with prediabetes (5, 6). Inadequate glycemic management in individuals with diabetes may increase their vulnerability to various diseases. Notably, prior studies have demonstrated a higher incidence of diabetes mellitus in patients diagnosed with cryptococcal disease. Furthermore, the presence of diabetes mellitus has been linked to an elevated mortality rate in this patient population (7).

Herein, we present the case of a 43-year-old, non-HIV-diagnosed, concurrently diabetic male with CM who developed PIIRS following initial antifungal therapy.

## Case presentation

**Figure 1:**
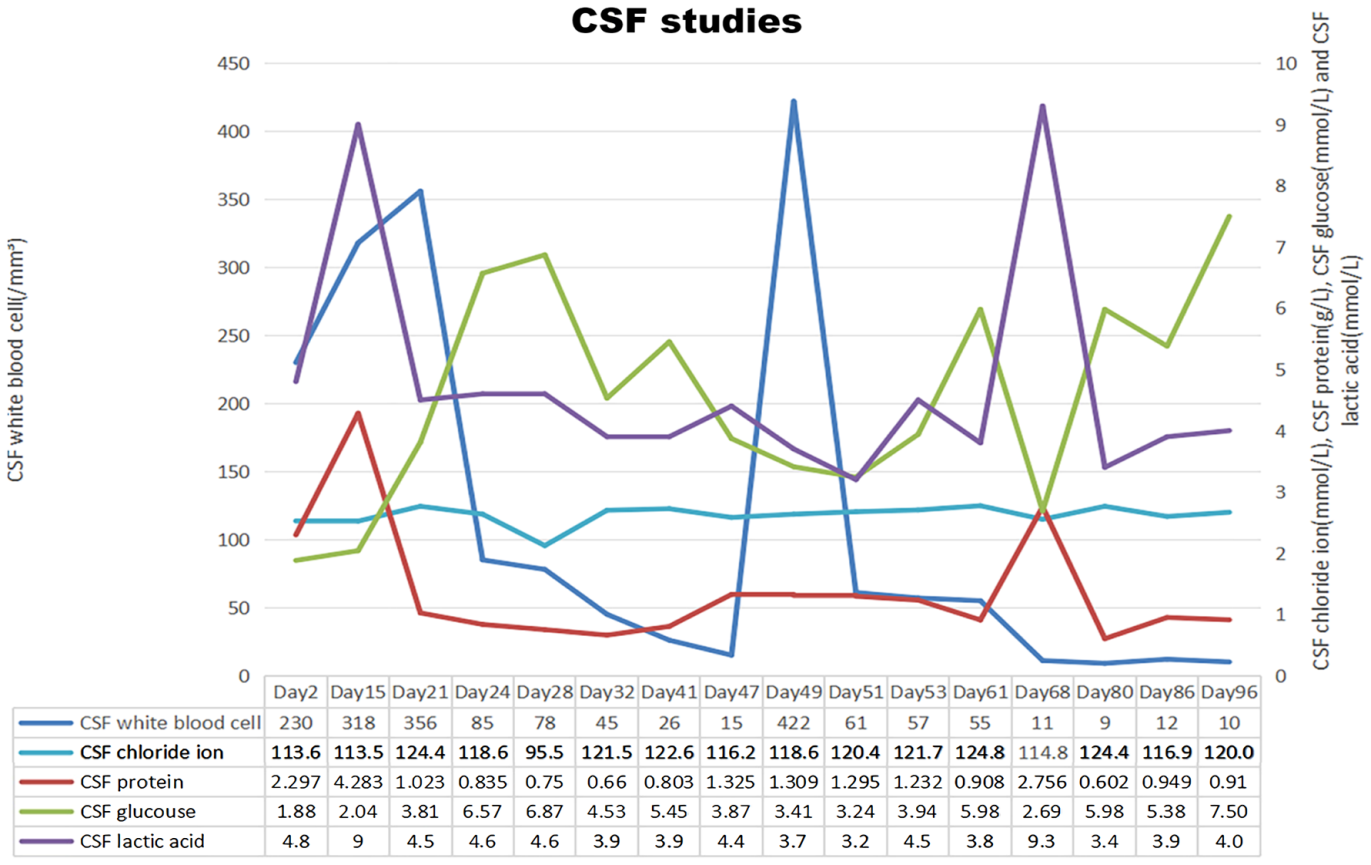
Chronologic representation of serial cerebrospinal fluid (CSF) measurements obtained from lumbar puncture with CSF white blood cell count on the left axis and CSF chloride ion, CSF protein, CSF glucose and CSF lactic acid on the right axis. In summary, the patient is demonstrating a gradual improvement across all assessments. An abnormal cerebrospinal fluid (CSF) white blood cell count observed on day 49, coupled with no substantial alterations in other metrics, indicates the possibility of a testing error. Conversely, the presence of abnormalities in all test parameters on day 68 suggests the potential onset of a new infection in the patient. (The standard reference ranges for leukocyte counts, chloride ion levels, protein concentration, glucose levels, and lactate in CSF are 0-5x10^6/L, 120-130mmol/L, 0.15∼0.45g/L, 2.5∼4.4mmol/L, 1.0∼2.8mmol/L.).

**Figure 2:**
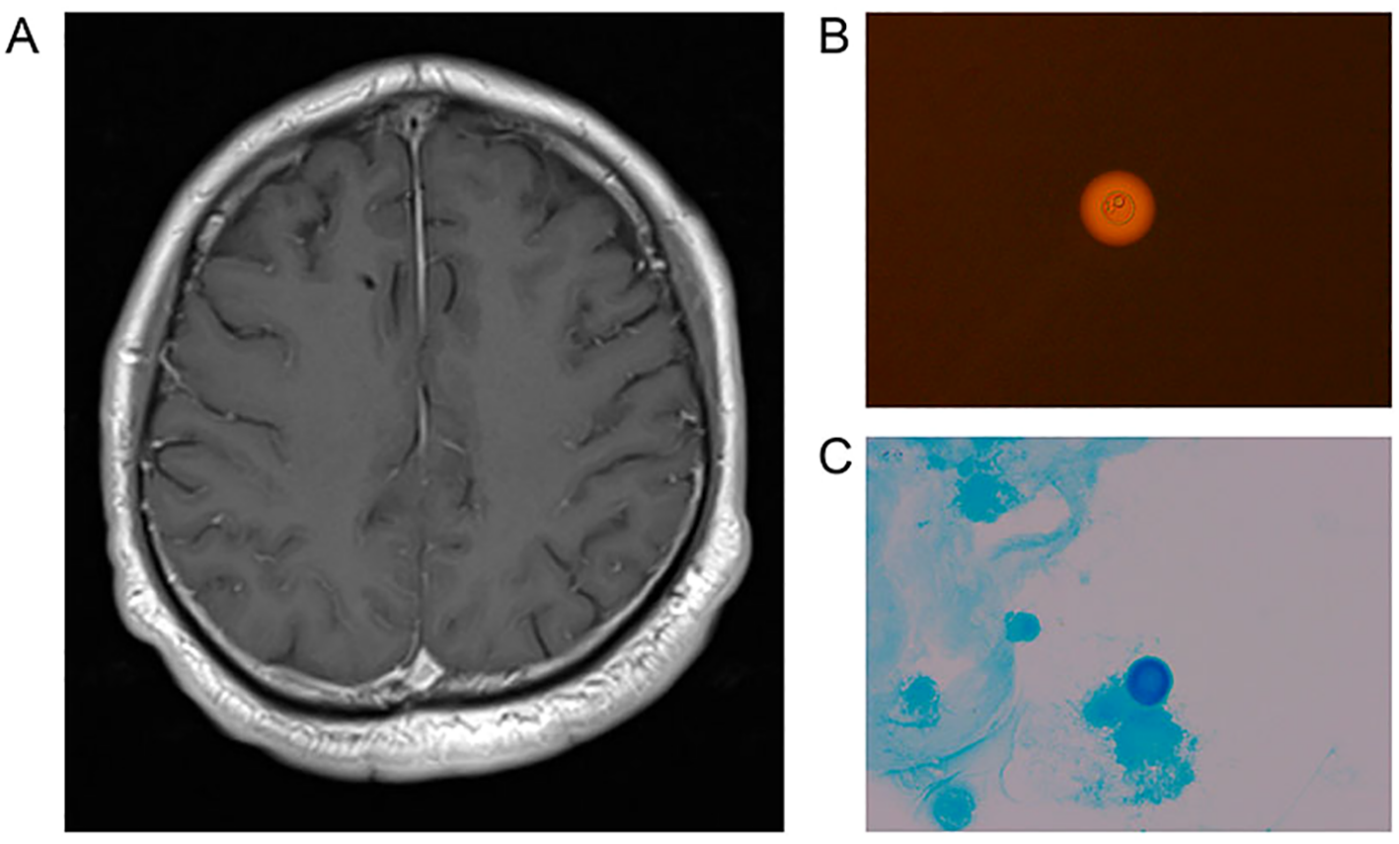
**(A)** MR imaging showed a marked meningeal enhancement on T1WI. Indicates the potential occurrence of cryptococcal meningoencephalitis in the patient. **(B, C)** The CSF result of India ink staining and Alcian blue staining of patients are both positive.

**Figure 3:**
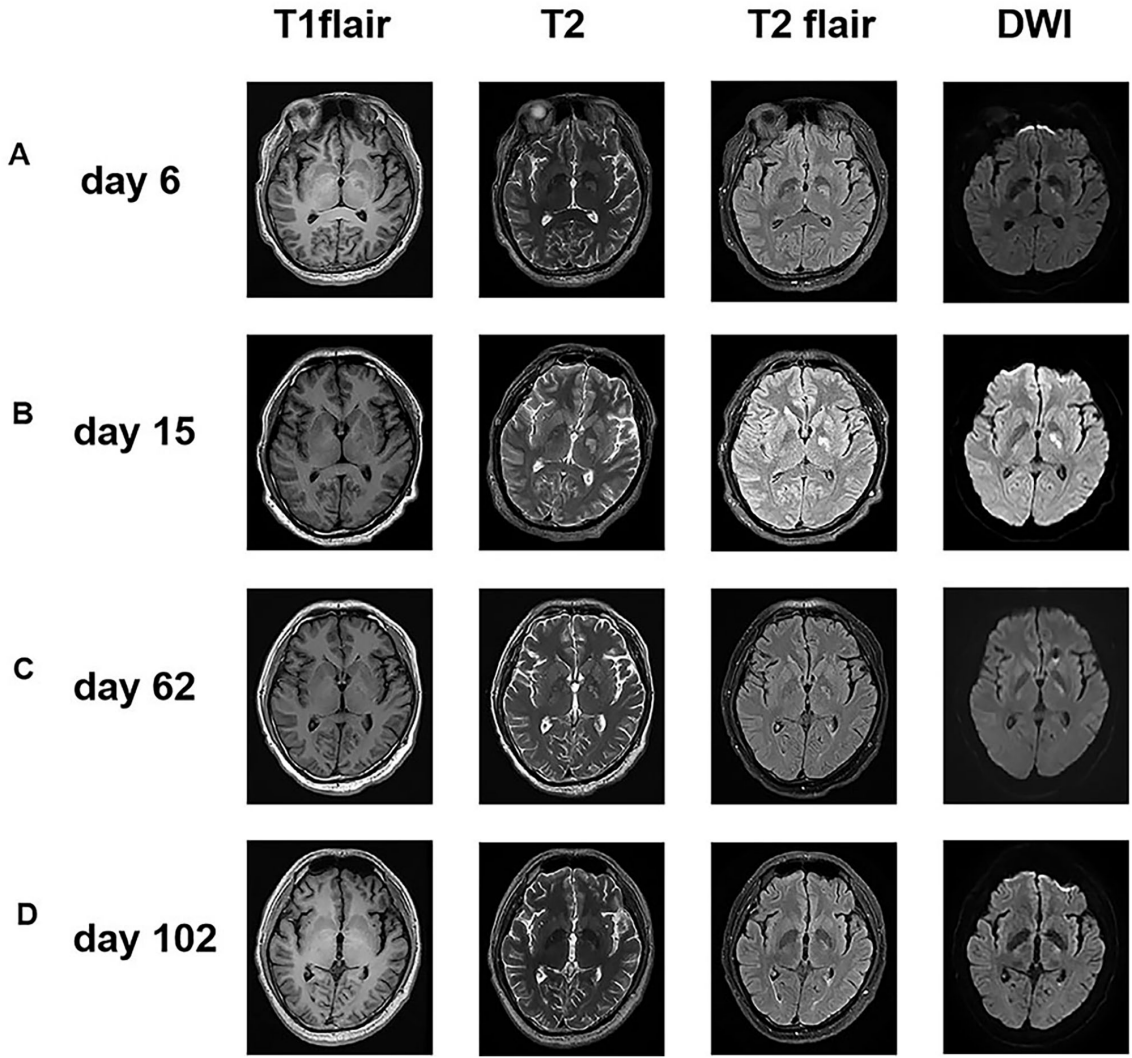
Serial axial brain MR imaging of the patient. **(A)** Imaging on Day 6 showed abnormal enhancement in left basal ganglia(T2/T2 Flair/DWI). **(B)** The imaging on Day 15 reveals the persistence of a lesion in the patient’s left basal ganglia prior to the omaya capsulotomy procedure. Furthermore, the images indicate that there are no contraindications to surgery in this patient. **(C)** Imaging on Day 62 showed marked improvement after effective antifungal therapy. The atypical signaling observed in the left basal ganglia is characterized by a notable reduction. **(D)** Imaging on Day 102 showed no marked lesion after corticosteroid treatment.

On 2023-07-17, a 47-year-old male was admitted to our hospital with a one-month history of headache and low-grade fever. He reported a ten-year history of diabetes mellitus characterized by poor glycemic control, without any history of trauma or surgery. Before admission to our hospital, he was diagnosed with viral encephalitis by lumbar puncture at an outside hospital, which showed an opening pressure(OP) (see **Figure 1**) of 380 mmH2O, white blood cell (WBC) count of 206/mm³, and an elevated CSF protein qualitative test; then he was treated with antiviral therapy and dehydration, which had an unsatisfactory effect, prompting his to seek further consultation at our hospital. Upon admission, he exhibited symptoms including fever (body temperature between 37.5°C to 38°C), headache for 25 days which aggravated in the past two days, accompanied by nausea and poor appetite. Physical examination revealed lethargy, depression, unresponsiveness, slight neck stiffness, a positive Kernig’s sign, and negative Babinski and Hoffman’s signs. Blood analysis revealed a WBC count of 6.06 × 10^9^/L (Neutrophils 76.1%, Lymphocytes 18.0%), C-reactive protein (CRP) 3.3 mg/L, and a negative serum HIV antibody test. A lumbar puncture revealed a high OP (see **Figure 1**) of 250 mmH2O, with CSF leukocyte count at 230/mm³ (23% neutrophils, 77% lymphocytes), protein levels of 2297 mg/L, glucose of 1.88 mmol/L, lactate of 4.8 mmol/L, and chloride of 113.6 mmol/L. India ink staining (8) and Alcian blue staining (9) of the CSF were both positive (see **Figures 2B, C**), with a positive cryptococcal polysaccharide antigen (CRAG) test. Subsequent (day 6) enhanced magnetic resonance (MR) imaging revealed involvement of the meninges (see **Figure 2A**) and basal ganglia (see **Figure 3A**). These findings led to a diagnosis of cryptococcal meningoencephalitis, prompting initiation of antifungal therapy with amphotericin B and 5-fluorocytosine(5-FC). The complete treatment regimen for amphotericin B extended over a duration of three months. Intravenous administration commenced at a dosage of 0.1 mg/kg per day, with a daily increment of 5 mg until reaching a maximum dosage of 0.7 mg/kg per day. The total cumulative dosage of the drug administered amounted to 3 grams. 5-Fluorocytosine is administered in the form of 0.25 g tablets, to be taken in four divided doses daily, over a treatment duration of two months. Post-treatment, intracranial pressure ranged from 250 mmH2O to 330 mmH2O, indicating refractory intracranial hypertension. Day 20, an Ommaya reservoir was placed in the right frontal area toward the ventricle for cranial pressure lowering therapy. A preoperative MRI was performed (see **Figure 3B**), and the findings are presented in the table. Intermittent postoperative fluid release via the Ommaya reservoir led to a gradual resolution of symptoms associated with high cranial pressure, including headache, nausea, and vomiting. On day 51 of admission, the patient exhibited sudden onset of headache, nausea, vomiting, and fever. An urgent lumbar puncture (see **Figure 1**) yielded cerebrospinal fluid with 422 leukocytes/mm³ (75% neutrophils, 12% lymphocytes), glucose levels of 3.41 mmol/L, protein at 1,325 mg/L, lactate at 3.7 mmol/L, and chloride at 118.6 mmol/L, with negative cryptococcal staining. Given these test results, PIIRS was not considered and an intracranial bacterial infection was suspected, with the possibility of an infection related to Ommaya reservoir. Administer 1 gram of ceftriaxone sodium via intravenous injection twice daily, resulting in a gradual improvement of symptoms. An MRI performed on the 62nd day after admission showed significant improvement in the patient’s lesions in the basal ganglia (see **Figure 3C**).

**Figure 4:**
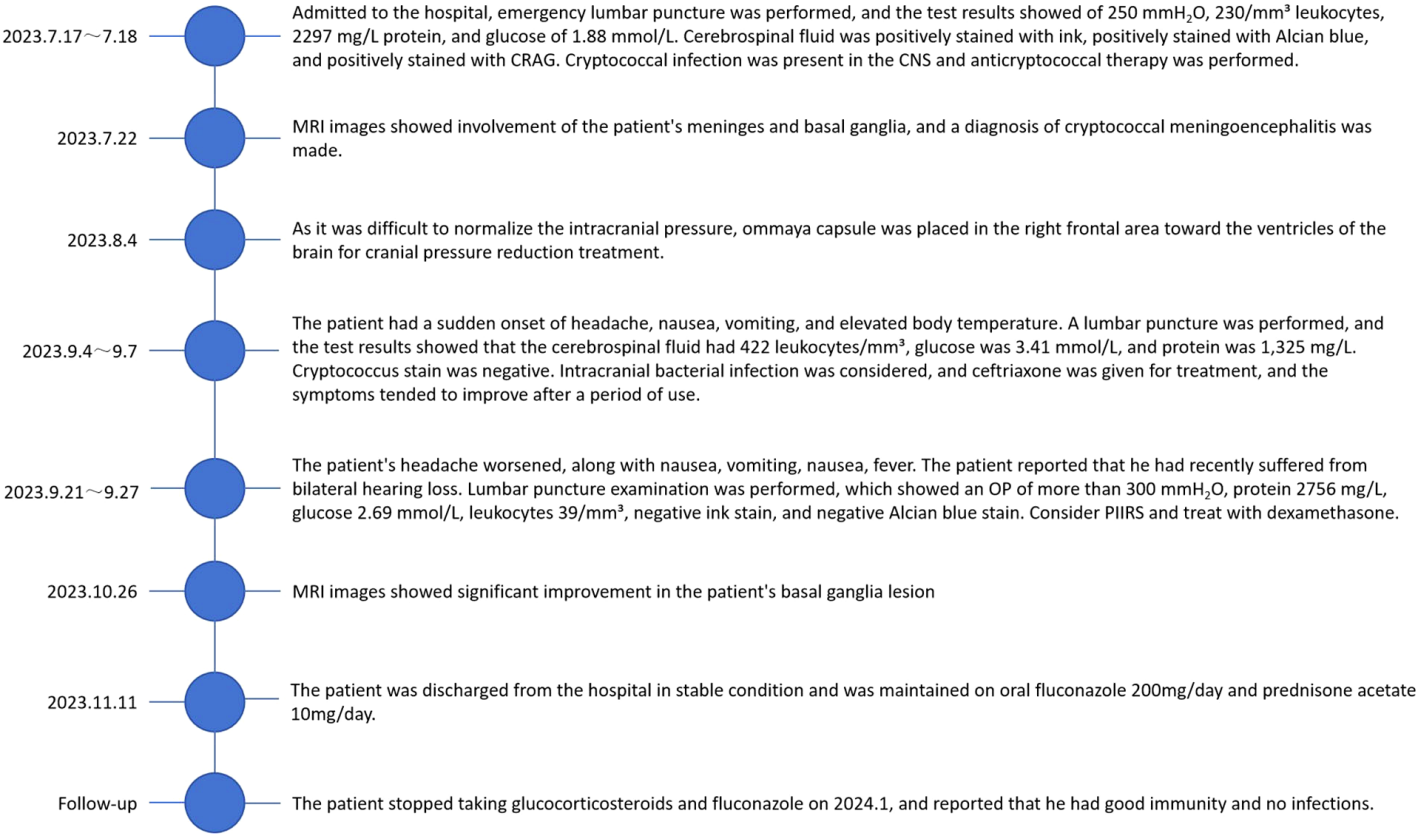
Graphical representation of the diagnose and therapy of the CM and PIIRS in our patient.

By the 67th day of admission, the patient experienced worsening headache, repeated nausea, vomiting, and fever. Initially, intracranial hypertension was suspected, prompting an increase in the frequency of cerebrospinal fluid extraction from every other day to daily, which yielded poor results. Subsequently, suspecting an adverse digestive reaction to 5-fluorocytosine (5-FC), the 5-FC was switched to fluconazole, 400mg IV daily; however, the patient’s symptoms did not significantly improve. Than the patient reported recent bilateral hearing loss. A lumbar puncture on the 71st day indicated an OP (see **Figure 1**) higher than 300 mmH2O, lactic acid of 9.3 mmol/L, protein of 2,756 mg/L, glucose of 2.69 mmol/L, and a leukocyte count of 39/mm³ (20% neutrophils and 68% lymphocytes), with a positive tryptophan test, and negative India ink and Alcian blue stains. Given these findings, PIIRS was considered, and treatment with 10 mg/d of dexamethasone was initiated. Following symptomatic improvement after 1 month, the dosage was gradually tapered and then transitioned to oral prednisone 30mg/d after four weeks. In light of the adverse effects associated with glucocorticoids, the dosage of glucose-lowering medications, including insulin, was modified accordingly. On days 89 and 96, the insulin was administered to sustain normoglycemia. On day 98, the patient received oral acarbose alongside a single subcutaneous injection of 16 units of glucagon, as well as three subcutaneous injections of mentholatum insulin, dosed at 4 units in the morning, 6 units in the afternoon, and 6 units in the evening. We dynamically adjust the insulin dosage based on the patient’s daily blood glucose level. The patient underwent a three-month therapy with amphotericin B combined with 5-FC, followed by a switch to oral fluconazole. MRI was performed on day 102 after admission (see **Figure 3D**) and showed that the patient’s brain lesions were reduced. Significant improvement in the patient’s clinical symptoms allowed for discharge in a stable condition on maintenance therapy of oral fluconazole (400 mg/day) and oral prednisone acetate (30 mg/day), with plans for regular follow-up visits for dosage adjustments based on therapeutic response. By day 110, a tapering of cortisol was initiated, which was accompanied by a corresponding reduction in insulin dosage. During follow-up visits, it was noted that the patient discontinued glucocorticoids around January 2024, exhibited poor glycemic control (approximately 10 mmol/L), but reported fair immunity without any infections. We have recorded a comprehensive account of the patient's entire hospitalization (**Figure 4**).

## Discussion

Cryptococcus, a capsulated fungus, primarily infects humans through the respiratory tract or skin, with species including *C. neoformans*, *C. deneoformans*, and *C. gattii* complex being pathogenic to humans (10). Among these, *C. neoformans* is predominantly responsible for cryptococcal meningitis in DM patients, typically observed in immunocompromised individuals, particularly those living with HIV (11). However, due to the administration of highly active antiretroviral therapy (HAART) in recent years, cryptococcal infections are increasingly seen in non-HIV-diagnosed immunocompromised patients, such as solid organ transplant(SOT) recipients, DM patients, and SLE patients. In individuals with DM, hyperglycemia creates an environment conducive to the proliferation of microorganisms, including Cryptococcus. DM patients with poorly controlled blood glucose levels impair the body’s level of cellular immunity, making the body less immune and more susceptible to cryptococcus (12). Consequently, DM patients with CM exhibit a marked increase in disease severity and mortality rates (11). Cryptococcus exhibits a pronounced affinity for the central nervous system, with the potential to progress from asymptomatic cryptococcal antigenemia to symptomatic cryptococcal meningoencephalitis. Additionally, Cryptococcus can affect various other organ systems, manifesting as nodules, hilar lymphadenopathy, and pulmonary calcifications, as well as osteolytic lesions in the bones and molluscum contagiosum lesions in the skin (13). In this case we report an non-HIV-diagnosed CM patient with ten years of poorly controlled DM who was hospitalized for more than reached four months.

Delay in diagnosis is a significant factor contributing to the suboptimal recovery from cryptococcal infection (14). Consequently, prompt diagnosis of the disease can lead to improved patient outcomes, which is informed by clinical presentation, laboratory findings and imaging results. The clinical presentation of CM varies significantly with the immune status of the host (15). In individuals with no significant impairment of immune function, headache is the most common clinical manifestation, and fever is less common (16). For the group with fever, the simultaneous presence of markedly elevated intracranial pressure suggests a poorer prognosis for the patient (17). Additionally, patients may exhibit decreased visual acuity (18), diplopia (19), and either unilateral or bilateral hearing impairment (20). In immunocompromised individuals, symptoms tend to be more insidious and varied, ranging from a single nodule to widespread dissemination within the organism (21). Currently, the culture test is considered the gold standard for laboratory detection of cryptococcal infection (22). However, given the prolonged duration required for results, the CrAg LFA is now regarded as the most applicable laboratory test available (23). Support for the diagnosis of cryptococcal meningoencephalitis came from an enhanced MRI conducted on 2023-9-16, revealing involvement of the meninges and basal ganglia. India ink stain is also viable in settings where local healthcare facilities lack resources. MRI is recommended for neuroimaging in patients with CM, but it is usually of low sensitivity and specificity, and requires a combination of clinical presentation and laboratory results to make a definitive diagnosis (21). In the reported case, diagnostic delay was attributed to the nonspecific symptoms such as headache and low-grade fever. Cryptococcal infection of the central nervous system was confirmed through positive India ink, Alisin blue, and cryptococcal antigen tests in cerebrospinal fluid samples at our hospital (TABLE).

The 2010 IDSA treatment guidelines categorize the treatment of non-HIV-diagnosed, non-SOT CM patients into three distinct phases: induction, consolidation, and maintenance, with amphotericin B(AmB)+5-FC for 4-6 weeks in the induction phase, fluconazole for 8 weeks in the consolidation phase, and fluconazole for 6-12 months in the maintenance phase (24). AmB’s input-related toxicity can cause headache, nausea, vomiting, and renal impairment (25), with decreased renal function leading to an accumulation of fluconazole, thereby exacerbating its toxicity (26). Thus, closely monitoring the patient’s condition during AmB treatment is crucial. In our case, severe nausea and vomiting of unknown origin, along with poor natriuresis, manifested during induction therapy, initially leading to suspicions of fluconazole toxicity as the cause. A brief transition from fluconazole to fluconazole proved ineffective, conclusively ruling out adverse reactions attributable to fluconazole toxicity in this instance. Furthermore, statistical evidence indicates that CM patients with DM exhibit higher intracranial pressure compared to those without DM (27). Methods for reducing intracranial pressure in CM patients include daily lumbar puncture, temporary lumbar drainage, ventriculoperitoneal shunting (VPS), and the use of an Ommaya reservoir and so on (28). The combination of triple antifungal therapy and VPS has been shown to be more effective than lumbar puncture alone in treating CM patients (29). However, on the one hand, the long-term effect of VPS is not as good as general conservative treatment (30), and on the other hand, it is also prone to postoperative complications such as localized infections (31). Recent data indicate that VPS usage may correlate with PIIRS, the grave complication in CM treatment (32). The Ommaya reservoir enables both the extraction of cerebrospinal fluid to mitigate intracranial pressure and the direct injection of antifungal drugs into the cerebrum, enhancing the drug concentration within the cerebrospinal fluid (28, 33). Consequently, employing the Ommaya reservoir for CM patients with elevated intracranial pressure could surpass VPS in fulfilling the long-term clinical requirements of these patients.

PIIRS is defined as a deterioration in the mental status of non-HIV-diagnosed CM patients following appropriate antifungal therapy, until CSF fungal cultures test negative (2). Hyperglycemia, which is a consequence of diabetes, leads to the dysfunction of macrophages and impairs the synthesis of tumor necrosis factor. This condition renders individuals with diabetes particularly vulnerable to fungal infections, notably cryptococcal infections (34). The causation of PIIRS may be linked to biased Th1 cell formation and impaired T-cell-macrophage differentiation (35, 36). In Type II diabetes and obesity, prolonged metabolic disturbances and inflammatory responses result in lymphocyte Th1 dominance and excessive activation of M2 macrophages (37, 38). The impact of stable diabetes on immune and inflammatory responses might contribute to the development of PIIRS. Concurrently, diabetes-induced immune abnormalities, which elevate the body’s cryptococcal burden, may also play a part in PIIRS development (39). Since the occurrence of PIIRS has a negative impact on the healing process of patients, timely diagnosis and appropriate treatment are crucial. As for clinical symptoms, hearing impairment as well as high intracranial pressure (especially >330 mmH2O) are high-risk predictors of PIIRS (32). Among laboratory tests, the baseline neutrophil ratio in CSF, neutrophil count, IgM, IL-6, and d-dimer may be predictive of the occurrence of PIIRS (39). Although the reliability of the above indexes has yet to be tested due to sample size limitations, an attempt can be made to include some or all of these indexes in the monitoring of PIIRS, considering the adverse effects of PIIRS on the long-term healing of patients. There are currently two main therapeutic regimens for PIIRS: (1) intravenous methylprednisolone 1 g for 5-7 days followed by a gradual dosage taper and (2) oral dexamethasone 10-20 mg/kg/day or intravenous prednisone 1 mg/kg/day and then tapered as appropriate (40). Determining which method is more effective requires further research. In the case reported herein, the second method was implemented, and significant symptom improvement was observed, followed by continued maintenance therapy with glucocorticoids.

## Conclusion

Timely diagnosis and treatment of CM can effectively decelerate the disease’s progression and enhance the prognosis. DM with poorly controlled blood glucose levels is associated with decreased immune function and a disturbed inflammatory response. This results in more severe disease, prolonged duration of illness and greater susceptibility to complications in patients with CM. Effective blood glucose management in DM patients diminishes the risk of cryptococcal infection as well as the incidence of complications. For CM patients experiencing elevated cranial pressure, the Ommaya reservoir may yield superior outcomes compared to VPS. In CM patients with DM, vigilance for the potential development of PIIRS is crucial, necessitating the integration of clinical symptoms with laboratory findings to facilitate early diagnosis and effective treatment.

## Data Availability

The raw data supporting the conclusions of this article will be made available by the authors, without undue reservation.

## References

[1] Chowdhary A Rhandhawa HS Prakash A Meis JF. Environmental prevalence of Cryptococcus neoformans and Cryptococcus gattii in India: an update. Crit Rev Microbiol. (2012) 38(1):1–16. doi: 10.3109/1040841X.2011.606426 22133016

[2] Williamson PR Jarvis JN Panackal AA Fisher MC Molloy SF Loyse A. Cryptococcal meningitis: epidemiology, immunology, diagnosis and therapy. Nat Rev Neurol. (2017) 13:13–24. doi: 10.1038/nrneurol.2016.167 27886201

[3] Haddow LJ Colebunders R Meintjes G Lawn SD Elliott JH Manabe YC. Cryptococcal immune reconstitution inflammatory syndrome in HIV-1-infected individuals: proposed clinical case definitions. Lancet Infect Dis. (2010) 10:791–802. doi: 10.1016/S1473-3099(10)70170-5 21029993 PMC3026057

[4] Anjum S Dean O Kosa P Magone MT King KA Fitzgibbon E. Outcomes in previously healthy cryptococcal meningoencephalitis patients treated with pulse taper corticosteroids for post-infectious inflammatory syndrome. Clin Infect Dis. (2021) 73:e2789–98. doi: 10.1093/cid/ciaa1901 PMC8563180 33383587

[5] Chan JCN Zhang Y Ning G. Diabetes in China: a societal solution for a personal challenge. Lancet Diabetes Endocrinol. (2014) 2:969–79. doi: 10.1016/S2213-8587(14)70144-5 25218728

[6] Xu Y Wang L He J Bi Y Li M Wang T. Prevalence and control of diabetes in Chinese adults. JAMA. (2013) 310:948–59. doi: 10.1001/jama.2013.168118 24002281

[7] Lin KH Chen CM Chen TL Kuo SC Kao CC Jeng YC. Diabetes mellitus is associated with acquisition and increased mortality in HIV-uninfected patients with cryptococcosis: A population-based study. J Infect. (2016) 72:608–14. doi: 10.1016/j.jinf.2016.01.016 26920792

[8] Rajasingham R Wake RM Beyene T Katende A Letang E Boulware DR. Cryptococcal meningitis diagnostics and screening in the era of point-of-care laboratory testing. J Clin Microbiol. (2019) 57(1):e01238-18. doi: 10.1128/JCM.01238-18 30257903 PMC6322457

[9] Adam P Sobek O Dolezil D Lodin Z Kasík J Hajduková L. Cryptococcal meningitis–a follow-up study of a serious clinical entity: quick cytological and microbiological diagnostics using a special staining procedure in cerebrospinal fluid specimens. Folia Microbiol (Praha). (2009) 54:567–8. doi: 10.1007/s12223-009-0084-8 20140729

[10] Freij JB Fu MS De Leon Rodriguez CM Dziedzic A Jedlicka AE Dragotakes Q. Conservation of intracellular pathogenic strategy among distantly related cryptococcal species. Infect Immun. (2018) 86:e00946–17. doi: 10.1128/IAI.00946-17 PMC6013651 29712729

[11] Nsenga L Kajjimu J Olum R Ninsiima S Kyazze AP Ssekamatte P. Cryptococcosis complicating diabetes mellitus: a scoping review. Ther Adv Infect. (2021) 8:204993612110147. doi: 10.1177/20499361211014769 PMC8111545 33996076

[12] Daryabor G Atashzar MR Kabelitz D Meri S Kalantar K. The effects of type 2 diabetes mellitus on organ metabolism and the immune system. Front Immunol. (2020) 11:1582. doi: 10.3389/fimmu.2020.01582 32793223 PMC7387426

[13] Meya DB Williamson PR. Cryptococcal disease in diverse hosts. N Engl J Med. (2024) 390:1597–610. doi: 10.1056/NEJMra2311057 38692293

[14] Aye C Henderson A Yu H Norton R. Cryptococcosis—the impact of delay to diagnosis. Clin Microbiol Infect. (2016) 22:632–5. doi: 10.1016/j.cmi.2016.04.022 27172806

[15] Zunt JR Baldwin KJ. Chronic and subacute meningitis. Continuum (Minneap Minn). (2012) 18:1290–318. doi: 10.1212/01.CON.0000423848.17276.21 23221842

[16] Marr KA Sun Y Spec A Lu N Panackal A Bennett J. A multicenter, longitudinal cohort study of cryptococcosis in human immunodeficiency virus–negative people in the United States. Clin Infect Dis. (2020) 70:252–61. doi: 10.1093/cid/ciz193 PMC6938979 30855688

[17] Ssebambulidde K Anjum SH Hargarten JC Chittiboina P Shoham S Seyedmousavi S. Treatment recommendations for non-HIV associated cryptococcal meningoencephalitis including management of post-infectious inflammatory response syndrome. Front Neurol. (2022) 13:994396. doi: 10.3389/fneur.2022.994396 36530631 PMC9751747

[18] Liebman DL Tam EK Lithgow MY Kane JE Fischbein NJ Lefebvre DR. Optic perineuritis associated with cryptococcal meningitis presenting with a “Hot orbit” in a patient with chronic lymphocytic leukemia. J Neuro-Ophthalmol. (2022) 42:272–7. doi: 10.1097/WNO.0000000000001538 PMC9124683 35421041

[19] Romani L Williamson PR Di Cesare S Di Matteo G De Luca M Carsetti R. Cryptococcal meningitis and post-infectious inflammatory response syndrome in a patient with X-linked hyper igM syndrome: A case report and review of the literature. Front Immunol. (2021) 12:708837. doi: 10.3389/fimmu.2021.708837 34335625 PMC8320724

[20] King KA Ansari G Panackal AA Zalewski C Anjum S Bennett JE. Audiologic and otologic complications of cryptococcal meningoencephalitis in non-HIV previously healthy patients. Otol Neurotol. (2019) 40:e657–64. doi: 10.1097/MAO.0000000000002242 PMC6565454 31157723

[21] Essien F Westbrook M Wolfley G Patterson S Carrol M. [amp]]lsquo;When *Cryptococcus* strikes and lupus is found’: a unique covert unveiling of systemic lupus erythematosus presenting as subacute meningitis. Ther Adv Chronic Dis. (2022) 13:204062232211027. doi: 10.1177/20406223221102784 PMC9280844 35847478

[22] Kiertiburanakul S Wirojtananugoon S Pracharktam R Sungkanuparph S. Cryptococcosis in human immunodeficiency virus-negative patients. Int J Infect Dis. (2006) 10:72–8. doi: 10.1016/j.ijid.2004.12.004 16288998

[23] Boulware DR Rolfes MA Rajasingham R von Hohenberg M Qin Z Taseera K. Multisite validation of cryptococcal antigen lateral flow assay and quantification by laser thermal contrast. Emerg Infect Dis. (2014) 20:45–53. doi: 10.3201/eid2001.130906 24378231 PMC3884728

[24] Perfect JR Dismukes WE Dromer F Goldman DL Graybill JR Hamill RJ. Clinical practice guidelines for the management of cryptococcal disease: 2010 update by the infectious diseases society of america. Clin Infect Dis. (2010) 50:291–322. doi: 10.1086/649858 20047480 PMC5826644

[25] Hamill RJ. Amphotericin B formulations: a comparative review of efficacy and toxicity. Drugs. (2013) 73:919–34. doi: 10.1007/s40265-013-0069-4 23729001

[26] Ngan NTT Flower B Day JN. Treatment of cryptococcal meningitis: how have we got here and where are we going? Drugs. (2022) 82:1237–49. doi: 10.1007/s40265-022-01757-5 PMC9483520 36112342

[27] Li H Li X Zhang L Fang W Zhang K Arastehfar A. The clinical profiles and outcomes of HIV-negative cryptococcal meningitis patients in type II diabetes mellitus. BMC Infect Dis. (2021) 21:224. doi: 10.1186/s12879-021-05867-5 33639846 PMC7913410

[28] Wan Y Li X Wang Y Yu Y Yang S. Clinical characteristic of 15 cases of cryptococcal meningitis treated with Ommaya reservoir. Acta Neurol Belg. (2020) 120:1139–45. doi: 10.1007/s13760-019-01193-5 31321616

[29] Li M Liu J Deng X Gan Q Wang Y Xu X. Triple therapy combined with ventriculoperitoneal shunts can improve neurological function and shorten hospitalization time in non-HIV cryptococcal meningitis patients with increased intracranial pressure. BMC Infect Dis. (2020) 20:844. doi: 10.1186/s12879-020-05510-9 33198666 PMC7667777

[30] Wen J Yin R Chang J Chen Y Dong X Cao W. Short-term and long-term outcomes in patients with cryptococcal meningitis after ventriculoperitoneal shunt placement. Front Neurol. (2022) 13:773334. doi: 10.3389/fneur.2022.773334 36468057 PMC9712185

[31] Xu L Zhu J Wang X Zeng G Gao Z Liu J. Clinical features and risk factors of surgical site infections in HIV-negative patients with cryptococcal meningitis underwent ventriculoperitoneal shunt operations: a retrospective study. BMC Infect Dis. (2022) 22:736. doi: 10.1186/s12879-022-07719-2 36104794 PMC9476323

[32] Liu J Li M Gan Zq Wang Yj Lin Cr Chen Zl. Postinfectious inflammatory response syndrome in HIV-uninfected and nontransplant men after cryptococcal meningitis. Future Microbiol. (2020) 15:613–21. doi: 10.2217/fmb-2019-0252 32490698

[33] Wei B Qian C Liu Y Lin X Wan J Wang Y. Ommaya reservoir in the treatment of cryptococcal meningitis. Acta Neurol Belg. (2017) 117:283–7. doi: 10.1007/s13760-016-0682-6 27492153

[34] Tsai ST Lin FY Chen PS Chiang HY Kuo CC. Three-year mortality in cryptococcal meningitis: Hyperglycemia predict unfavorable outcome. PloS One. (2021) 16(5):e0251749. doi: 10.1371/journal.pone.0251749 34048463 PMC8162582

[35] Panackal AA Wuest SC Lin YC Wu T Zhang N Kosa P. Paradoxical immune responses in non-HIV cryptococcal meningitis. PloS Pathog. (2015) 11:e1004884. doi: 10.1371/journal.ppat.1004884 26020932 PMC4447450

[36] Williamson PR. Post-infectious inflammatory response syndrome (PIIRS): Dissociation of T-cell-macrophage signaling in previously healthy individuals with cryptococcal fungal meningoencephalitis. Macrophage (Houst). (2015) 2:e1078. doi: 10.14800/Macrophage.1078 27064474 PMC4825797

[37] Cheng X Wang J Xia N Yan XX Tang TT Chen H. A guanidine-rich regulatory oligodeoxynucleotide improves type-2 diabetes in obese mice by blocking T-cell differentiation. EMBO Mol Med. (2012) 4:1112–25. doi: 10.1002/emmm.201201272 PMC3491840 23027613

[38] Feuerer M Herrero L Cipolletta D Naaz A Wong J Nayer A. Lean, but not obese, fat is enriched for a unique population of regulatory T cells that affect metabolic parameters. Nat Med. (2009) 15:930–9. doi: 10.1038/nm.2002 PMC3115752 19633656

[39] Wang Y Wei H Shen L Su X Liu J Xu X. Immunological predictors of post infectious inflammatory response syndrome in HIV-negative immunocompetent cryptococcal meningitis. Front Immunol. (2022) 13:895456. doi: 10.3389/fimmu.2022.895456 35686135 PMC9171325

[40] Liu J Liu J Qin B e Yao S Wang A Yang L. Post-infectious inflammatory response syndrome in an HIV-negative immunocompetent elderly patient with cryptococcal meningitis: A case report and literature review. Front Immunol. (2022) 13:823021. doi: 10.3389/fimmu.2022.823021 35281037 PMC8904365

